# Multi-trait genomic prediction using in-season physiological parameters increases prediction accuracy of complex traits in US wheat

**DOI:** 10.1186/s12864-022-08487-8

**Published:** 2022-04-12

**Authors:** Dipendra Shahi, Jia Guo, Sumit Pradhan, Jahangir Khan, Muhsin AVCI, Naeem Khan, Jordan McBreen, Guihua Bai, Matthew Reynolds, John Foulkes, Md Ali Babar

**Affiliations:** 1 Department of Agronomy, 3105 McCarty Hall B, Gainesville, FL 32611 USA; 2 Department of Forest Ecosystem and Society, Oregon State University, 3180 SW Jefferson Way, Corvallis, OR 97331 USA; 3 USDA-ARS, Manhattan, KS USA; 4 CIMMYT International Maize and Wheat Improvement Center (CIMMYT), Km. 45, Carretera Mexico, El Batan, Texcoco, Mexico; 5 Division of Plant and Crop Sciences, School of Biosciences, University of Nottingham, Leicestershire, LE12 5RD UK

**Keywords:** Canopy temperature, NDVI, Genomic prediction, Multi-trait genomic prediction, Spike partitioning index, Fruiting efficiency

## Abstract

**Background:**

Recently genomic selection (GS) has emerged as an important tool for plant breeders to select superior genotypes. Multi-trait (MT) prediction model provides an opportunity to improve the predictive ability of expensive and labor-intensive traits. In this study, we assessed the potential use of a MT genomic prediction model by incorporating two physiological traits (canopy temperature, CT and normalized difference vegetation index, NDVI) to predict 5 complex primary traits (harvest index, HI; grain yield, GY; grain number, GN; spike partitioning index, SPI; fruiting efiiciency, FE) using two cross-validation schemes CV1 and CV2.

**Results:**

In this study, we evaluated 236 wheat genotypes in two locations in 2 years. The wheat genotypes were genotyped with genotyping by sequencing approach which generated 27,466 SNPs. MT-CV2 (multi-trait cross validation 2) model improved predictive ability by 4.8 to 138.5% compared to ST-CV1(single-trait cross validation 1). However, the predictive ability of MT-CV1 was not significantly different compared to the ST-CV1 model.

**Conclusions:**

The study showed that the genomic prediction of complex traits such as HI, GN, and GY can be improved when correlated secondary traits (cheaper and easier phenotyping) are used. MT genomic selection could accelerate breeding cycles and improve genetic gain for complex traits in wheat and other crops.

**Supplementary Information:**

The online version contains supplementary material available at 10.1186/s12864-022-08487-8.

## Background

Phenotypic selection is widely used in most of the conventional plant breeding programs. However, this method is both labor and time-intensive as it involves screening for traits of interest across several years and environments [1–3]. Marker-assisted selection (MAS) has become an important part of modern breeding programs. It conventionally uses few molecular markers or large-effect QTLs and is mostly useful for traits governed by a small number of major genes [4, 5]. Most traits of interest are complex and are controlled by many genes, and thus the application of MAS in a practical breeding program may not be successful while working with many quantitative traits [6, 7]. Genomic selection (GS) is an indirect selection approach that improves the accuracy of marker-assisted selection (MAS) by using genome-wide markers that can capture QTL with both large and small effects [8, 9]. Genomic selection builds a model using phenotypic and genotypic data from a set of breeding lines called training population (TP). The model is then used to estimate the genetic values called genomic estimated breeding value (GEBV) of a set of tested lines called validation population (VP) that only have genotypic data [1, 4, 10, 11]. Genomic selection decreases the breeding cycle by selecting the progeny in the early stages or before being tested in field experiments based on GEBV. The rapid advancement of next-generation sequencing (NGS) methods like genotype-by-sequencing (GBS) has made it feasible to identify and genotype many SNPs across the entire genome in many crops including wheat [6, 12]. Genomic selection will also likely increase gain per unit cost by reduced genotyping cost per data point and reduced number of lines to be phenotyped [4, 13]. As a result, GS is being implemented widely in breeding programs to improve genetic gain and expedite cultivar development by reducing cycles of selection [1, 14]. Prediction accuracy is estimated as a correlation between GEBV and the phenotypic value of a trait [13]. Prediction accuracy is influenced by various factors such as models used, the number of markers (marker density), QTL numbers, training population size (sample size), population structure and relatedness among individuals in TP and VP, and the heritability of a trait, etc. [1, 15, 16]. Several statistical models have been proposed and used to implement GS. The parametric methods include ridge regression best linear unbiased prediction (rrBLUP) [17], genomic best linear prediction (GBLUP) [18], least absolute shrinkage and selection operator (LASSO) [19], and Bayesian-based methods: Bayesian ridge regression (BRR) [20], Bayes A, Bayes B, and Bayesian LASSO [21]. Likewise, non-parametric methods include reproducing kernel Hilbert spaces regression (RKHS) [22], neural networks [23], and random forests [24]. There is variation in prediction accuracies due to differences in their assumptions and algorithms concerning the variances of complex traits [6].

Physiological traits (PT) such as normalized difference vegetation index (NDVI) and canopy temperature (CT) are indicative of stress-resilient genotypes with efficient photosynthesis and respiration processes [25, 26]. Previous studies have reported significant correlations of these traits with grain yield (GY). A negative correlation between CT and GY has been reported in wheat under terminal heat stress conditions [26, 27]. NDVI has also been shown to be associated with wheat GY in different environments [26, 28–31]. The development of high throughput phenotyping (HTP) platforms makes it possible to screen a large number of genotypes in a short time at an affordable cost [26, 28, 32]. These PTs are good candidates to be used as indirect selection tools to select superior genotypes with stress tolerance and high yield potential [26, 33, 34]. Multi-trait (MT) genomic prediction is a strategy that incorporates one or more secondary traits that correlate with the primary trait to predict the accuracy of selecting a primary trait [8, 35, 36]. If a trait of interest has low heritability, MT- GS can be used to take the advantage of correlated traits with higher heritability to increase the predictive ability of traits of interest [36, 37]. It is also very useful if correlated traits are easier and more cost-effective to be phenotyped than the primary traits [38]. In most plant breeding programs, breeders usually collect phenotypic data of several traits, which enables them to take advantage of information from correlated traits along with genotypic information [39]. MT-GS methods have recently been applied due to increased prediction accuracies when the correlated traits are incorporated into the model [36, 37, 40–42] and showed the improved predictive ability of GY in wheat by including physiological traits [42–45]. In addition to yield, MT-GS has also been used to improve the predictive ability of other traits such as grain end-use quality [46], dry matter yield and water-soluble carbohydrates [47], and baking quality [48]. The main objectives of this study were to compare the relative performance of ST and MT-GS models and determine whether incorporating in-season physiological traits (NDVI and CT) in prediction models can improve the predictive ability of primary traits including HI, GN, GY, FE, and SPI.

## Results

### Analysis of variance

A combined ANOVA showed significant genotypic and environmental effects on correlated secondary traits, CT and NDVI, but genotype-by-environment interaction (G × E) was not significant (Table 1). However, genotypic, environmental, and G × E effects on all other primary traits (HI, GY, GN, SPI, and FE) were significant.

**Table 1:** Mean squares of the combined analysis of variance across different environments for primary and secondary traits

Traits	Genotype G	Environment E	Interaction G X E
HI	0.00753^c^	1.33432^c^	0.00440^c^
GY	1604151^c^	284430102^c^	1125063^b^
GN	13979431^c^	1820498352^c^	10631742^a^
SPI	0.00419^c^	0.33176^c^	0.00334^c^
FE	1147.4^c^	8185.1^c^	1531.6^c^
NDVI	43.8^c^	12,681.6^c^	17.7
CT	1.7^a^	3733.3^c^	1.4

*HI* harvest index, *GY* grain yield in kg ha^− 1^, *GN* grain number m^− 2^, *SPI* spike partitioning index, *FE* fruiting efficiency in grains g^− 1^ of spike dry weight at anthesis+ 7 days, *NDVI* normalized difference vegetation index, *CT* canopy temperature in ^o^ C

^a^, ^b^, ^c^significant at 0.05, 0.005 and 0.001 levels, respectively

### Basic summary and heritability

**Fig. 1:**
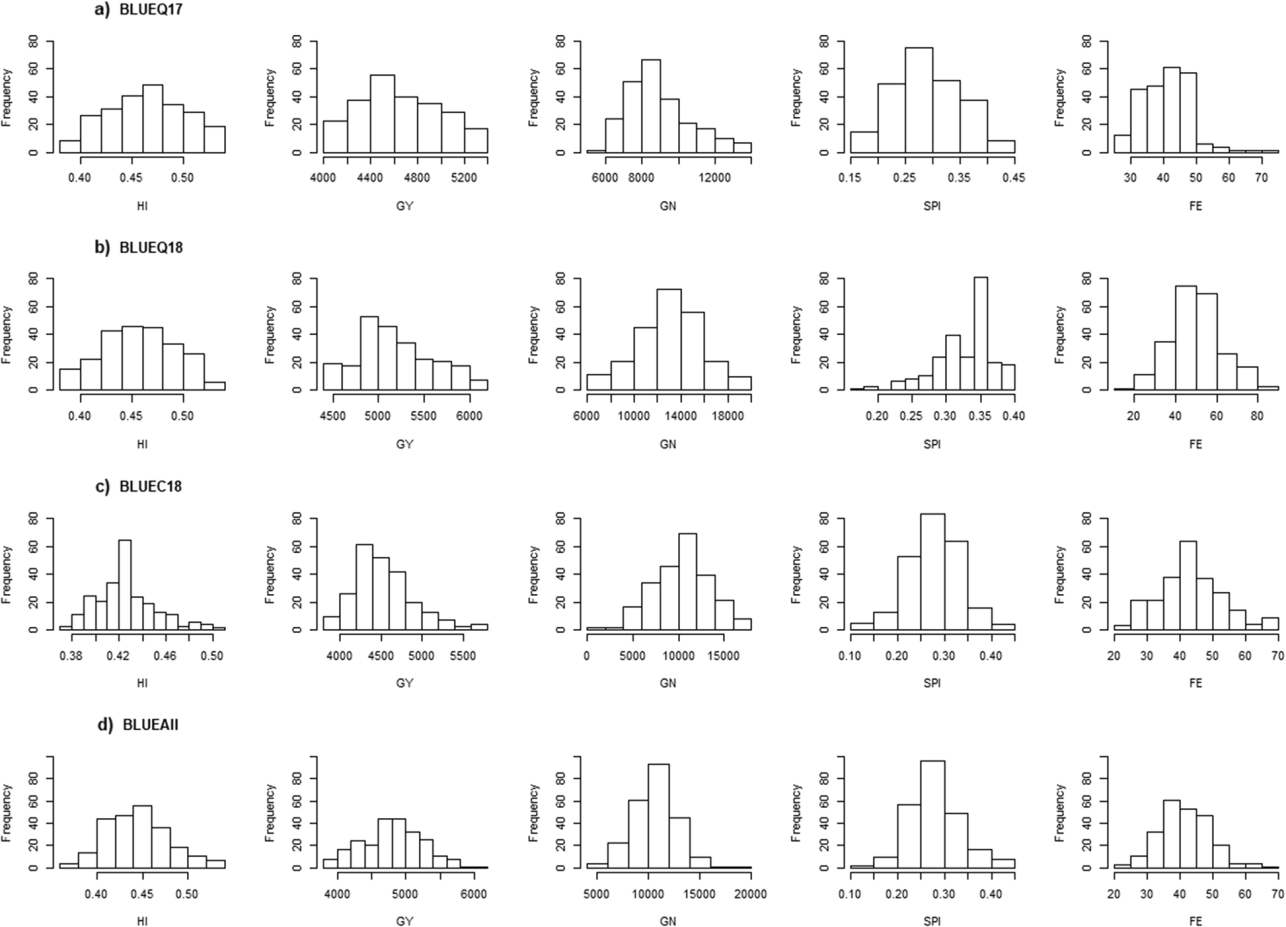
Distribution of adjusted means (BLUEs) for 5 traits in four datasets: **A** BLUEQ17, **B** BLUEQ18, **C** BLUEC18, and **D** BLUEAll

A wide range of variations for all traits was observed across all environments. The distribution of adjusted means (BLUEs) is shown in Fig. 1. The genotypes showed continuous variations for different traits. Table 2 lists the range, mean, standard deviation, and heritability for HI, GY, GN, SPI, FE, TGW, CT, and NDVI collected in different environments. The highest mean HI value was found in BLUEQ17 (0.46), and the lowest mean HI was found in BLUEC18 (0.42) (Table 2). The GY mean values ranged from 5047 kg ha^− 1^ (BLUEQ18) to 4483 kg ha^− 1^ (BLUEC18) (Table 2). Similarly, the mean values ranged from 8012 (BLUEQ17) to 13,212 (BLUEQ18) for GN, from 0.28 (BLUEC18) to 0.34 (BLUEQ18) for SPI, from 43.2 (BLUEC18) to 49.6 (BLUEQ18) grains g^− 1^ of spike dry weight at anthesis+ 7 days for FE (Table 2), from 0.58 (BLUEQ18) to 0.65 (BLUEC18) for NDVI, and from 26.7 (BLUEQ18) to 28.6 °C (BLUEC18) for CT (Table 2). The range of broad-sense heritability was large, with the highest for NDVI (0.64) followed by HI (0.38), CT (0.37), and GY (0.34), with the lowest for GN (0.28), SPI (0.26), and FE (0.25) (Table 2).

**Table 2:** Summary of adjusted means, range, standard deviation (SD), and heritability (H^2^) for phenotypic traits evaluated

	BLUEQ17			BLUEQ18			BLUEC18			BLUEAll			H^2^
Traits	Mean	Range	SD	Mean	Range	SD	Mean	Range	SD	Mean	Range	SD	
HI	0.46	0.41–0.53	0.06	0.44	0.40–0.52	0.07	0.42	0.37–0.5	0.07	0.44	0.37–0.53	0.05	0.38
GY	4650	4271–5338	676	5047	4490–6130	646	4483	3920–5714	604	4752	3920–6130	680	0.34
GN	8012	6018–13,597	2226	13,212	6050–18,807	2976	105,861	5338–17,411	3203	10,630	5338–18,807	2098	0.28
SPI	0.32	0.15–0.44	0.06	0.34	0.17–0.40	0.04	0.28	0.13–0.43	0.05	0.28	0.13–0.44	0.05	0.26
FE	44	26.5–72.9	9.4	49.6	24.5–72.4	13.8	43.2	24.5–93.3	11.7	41.6	24.1–68.3	7.7	0.25
NDVI	0.64	0.48–0.79	0.07	0.58	0.45–0.72	0.05	0.65	0.47–0.70	0.02	0.62	0.45–0.79	0.07	0.64
CT	27.7	25.1–30.2	1.4	26.7	24.5–31.5	1.14	28.6	23.3–32.7	1.8	27.1	23.0–32.7	0.54	0.37

*HI* harvest index, *GY* grain yield in kg ha^−1^, *GN* Grain number m^−2^, *SPI* spike partitioning index, *FE* fruiting efficiency in grains g^− 1^ of spike dry weight at anthesis+ 7 days, *NDVI* normalized difference vegetation index, *CT* canopy temperature in ^o^ C

### Phenotypic correlations

NDVI showed positive correlations with HI (0.14* to 0.41***), GY (0.30** to 0.46**), GN (0.20** to 0.42**), SPI (0.11 to 0.19**) and FE (0.09 to 0.16**), whereas CT showed negative correlations with HI (− 0.05 to − 0.23**), GY (− 0.15** to − 0.34**), GN (− 0.18** to − 0.37**), SPI (− 0.01 to − 0.21**) and FE (− 0.03 to − 0.14*) (Table 3). The correlation range was wide (0.09 to − 0.46***) between CT and NDVI. Harvest index had strong positive correlations with GY (0.50*** to 0.63***), GN (0.42*** to 0.50***), SPI (0.26** to 0.40**) and (FE 0.35** to 0.51***). Likewise, GY had positive and significant correlations with GN (0.76***-0.87***), SPI (0.08** to 0.34**) and FE (0.27** to 0.48***). GN also had significant positive correlations with SPI (0.06 to 0.38**) and FE (0.47*** to 0.54**). SPI was negatively correlated with FE (− 0.1 to − 0.50***).

**Table 3:** Pearson’s correlation coefficient between phenotypic traits by using best linear unbiased estimates in four datasets, A) BLUEQ17; B) BLUEQ18; C) BLUEC18; D) BLUEAll

Traits	HI	GY	GN	SPI	FE	NDVI	CT
A							
HI	1						
GY	0.61	1					
GN	0.47	0.87	1				
SPI	0.26	0.08	0.06	1			
FE	0.48	0.48	0.54	−0.50	1		
NDVI	0.18	0.30	0.39	0.19	0.16	1	
CT	−0.20	−0.26	− 0.37	− 0.19	−0.14	− 0.46	1
B							
HI	1						
GY	0.50	1					
GN	0.48	0.83	1				
SPI	0.33	0.28	0.21	1			
FE	0.47	0.38	0.47	−0.13	1		
NDVI	0.14	0.36	0.42	0.19	0.14	1	
CT	−0.05	−0.15	− 0.18	−0.01	− 0.03	−0.18	1
C							
HI	1						
GY	0.63	1					
GN	0.50	0.76	1				
SPI	0.40	0.34	0.38	1			
FE	0.51	0.27	0.52	−0.10	1		
NDVI	0.25	0.40	0.37	0.11	0.09	1	
CT	−0.23	−0.34	− 0.23	−0.21	− 0.03	−0.03	1
D							
HI	1						
GY	0.59	1					
GN	0.42	0.82	1				
SPI	0.29	0.25	0.23	1			
FE	0.35	0.32	0.47	−0.32	1		
NDVI	0.41	0.36	0.20	0.13	0.12	1	
CT	−0.11	−0.24	− 0.31	−0.07	− 0.08	0.09	1

*HI* harvest index, *GY* grain yield in kg ha^−1^, *GN* grain number m^−2^, *SPI* spike partitioning index, *FE* fruiting efficiency in grains g^− 1^ of spike dry weight at anthesis+ 7 days, *NDVI* normalized difference vegetation index, *CT* canopy temperature in ^o^C. Correlation coefficient value of above 0.14, 0.18 and 0.40 is significant at 0.001, 0.01, and 0.05 probability levels, respectively

### Principal component (PC) analysis

**Fig. 2:**
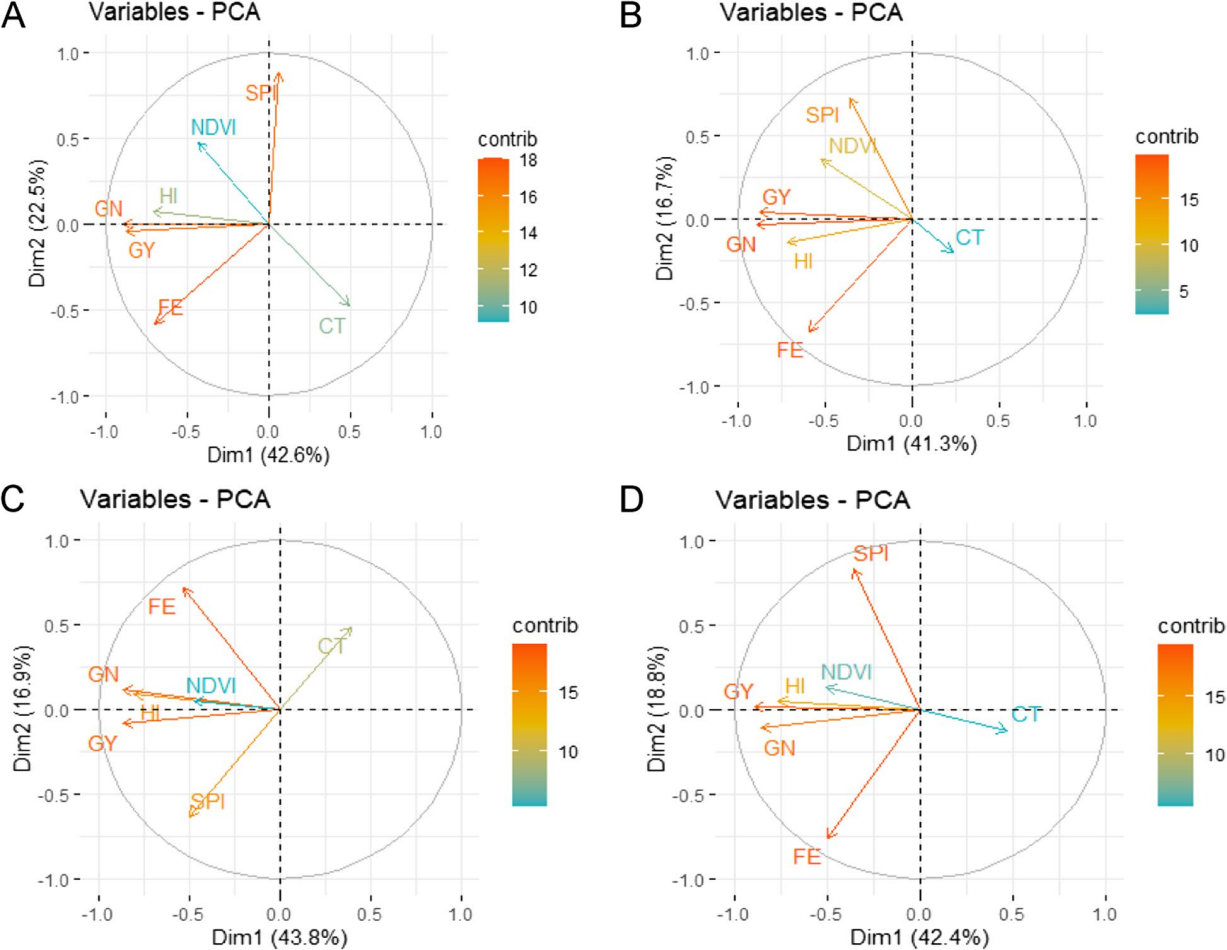
Principal component bi-plot analysis of measured traits using best linear unbiased estimates in four datasets: **A** BLUEQ17, **B** BLUEQ18, **C** BLUEC18, and **D** BLUEAll. HI, harvest index; GY, grain yield in kg ha^−1^; GN, grain number m^−2^; SPI, spike partitioning index; FE, fruiting efficiency in grains g^− 1^ of spike dry weight at anthesis+ 7 days; NDVI, normalized difference vegetation index; CT, canopy temperature in ^o^C

The PC biplots showed the first two PCs explained 65.1, 58, 60.7, 61.2% of the total variation in BLUEQ17, BLUEQ18, BLUEC18, and BLUEAll data, respectively (Fig. 2). It was observed that GY, GN, HI, and NDVI were mainly clustered together, which were distinctly separated from CT.

### Single-trait genomic prediction

**Fig. 3:**
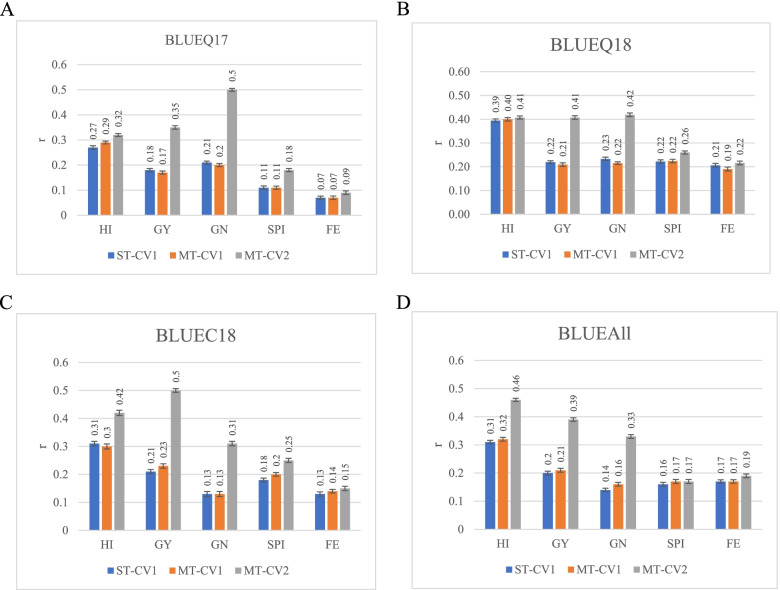
Bar graphs showing the predictive ability for 5 traits in four datasets: A BLUEQ17, B BLUEQ18, C BLUEC18, and D BLUEAll. Single-trait prediction model (ST-CV1), and multi-trait prediction mode (MT) with two schemes of cross-validation (MT-CV1 and MT-CV2. Mean Pearson’s correlations and standard error for each environment were presented for each trait. HI, harvest index; GY, grain yield in kg ha^− 1^; GN, grain number m^− 2^; SPI, spike partitioning index; FE, fruiting efficiency in grains g^− 1^ of spike dry weight at anthesis+ 7 days; NDVI, normalized difference vegetation index; CT, canopy temperature in ^o^C

Among the five traits evaluated by ST-CV1 model, the highest predictive ability was observed for HI (0.39) in BLUEQ18, and the lowest predictive ability was observed for FE (0.07) in BLUEQ17 (Table 4, Fig. 3). ST-CV1 predictive ability ranged from 0.27 (BLUEQ17) to 0.39 (BLUEQ18) for HI, from 0.18 (BLUEQ17) to 0.22 (BLUEQ18) for GY, from 0.13 (BLUEC18) to 0.23 (BLUEQ18) for GN, from 0.11 (BLUEQ17) to 0.22 (BLUEQ18) for SPI, from 0.07 (BLUEQ17) to 0.21(BLUEQ18) for FE. In general, the predictive abilities for GY, HI, and GN were higher than the partitioning traits SPI and FE.

**Table 4:** Table showing the predictive ability for 5 traits in four datasets

Locations	Traits	ST-CV1	MT-CV1	MT-CV2	% Increase from ST-CV1 to MT-CV2
BLUEQ17	HI	0.27	0.29	0.32	18.5
	GY	0.18	0.17	0.35	94.4
	GN	0.21	0.20	0.50	138.1
	SPI	0.11	0.11	0.18	63.6
	FE	0.07	0.07	0.09	28.6
BLUEQ18	HI	0.39	0.40	0.41	5.1
	GY	0.22	0.21	0.41	86.4
	GN	0.23	0.22	0.42	82.6
	SPI	0.22	0.22	0.26	18.2
	FE	0.21	0.19	0.22	4.8
BLUEC18	HI	0.31	0.30	0.42	35.5
	GY	0.21	0.23	0.50	138.1
	GN	0.13	0.13	0.31	138.5
	SPI	0.18	0.20	0.25	38.9
	FE	0.13	0.14	0.15	15.4
BLUEAll	HI	0.31	0.32	0.46	48.4
	GY	0.20	0.21	0.39	95.0
	GN	0.14	0.16	0.33	135.7
	SPI	0.16	0.17	0.17	6.3
	FE	0.17	0.17	0.19	11.8

Single-trait prediction model (ST-CV1), and multi-trait prediction mode (MT) with two schemes of cross-validation (MT-CV1 and MT-CV2); *HI* harvest index, *GY* grain yield in kg ha^−1^, *GN* grain number m^−2^, *SPI* spike partitioning index, *FE* fruiting efficiency in grains g^− 1^ of spike dry weight at anthesis+ 7 days, *NDVI* normalized difference vegetation index, *CT* canopy temperature in ^o^C

### Multi-trait cross-validation 1

In the MT-CV1 model, the predictive ability for the five primary traits was similar to that of the ST-CV1 and was not statistically significant (*p* > 0.05). In the MT-CV1 model, the predictive ability was highest for HI, from 0.29 (BLUEQ17) to 0.40 (BLUEQ18), but lowest for FE, from 0.07 (BLUEQ17) to 0.19 (BLUEQ18) (Table 4, Fig. 3). The value of MT-CV1 predictive ability for GN was ranged from 0.13 (BLUEC18) to 0.22 (BLUEQ18) (Table 4, Fig. 3). Likewise, MT-CV1 predictive ability for GY ranged from 0.17 (BLUEQ17) to 0.23 (BLUEC18) (Table 4, Fig. 3). For SPI, MT-CV1 predictive ability varied from 0.11 (BLUEQ17) to 0.22 (BLUEQ18) (Table 4, Fig. 3).

### Multi-trait cross-validation 2

The MT-CV2 included two physiological traits, NDVI and CT, as secondary traits on both training and validation sets. This in general improved the predictive ability for all traits (HI, GY, GN, FE, and SPI) compared to the models ST-CV1 and MT-CV1 with a single exception (Table 4, Fig. 3). The predictive ability for GY using the MT-CV2 model ranged from 0.35 (BLUEQ17) to 0.50 (BLUEC18) (Table 4, Fig. 3). For HI, MT-CV2 predictive ability ranged from 0.32 (BLUEQ17) to 0.46 (BLUEAll) (Table 4, Fig. 3). MT-CV2 predictive ability for GN differed from 0.31 (BLUEC18) to 0.50 (BLUEQ17). MT-CV2 predictive ability for FE was ranged from 0.09 (BLUEQ17) to 0.22 (BLUEQ18) (Table 4, Fig. 3). For SPI, MT-CV2 predictive ability varied from 0.17 (BLUEQAll) to 0.26 (BLUEQ18) (Table 4, Fig. 3).

When we compared ST-CV1 with MT-CV2, FE had the lowest percentage increase (5.1) in predictive ability (BLUEQ18), while GN showed the highest percentage increase of 138.5 (BLUEC18) in predictive ability (Table 4, Fig. 3). MT-CV2 model showed a better predictive ability than ST-CV1 with percentage increases from 82.6 (BLUEQ18) to 138.5 (BLUEC18, Fig. 3) for GN, from 5.1 (BLUEQ18) to 48.4 (BLUEAll) for HI, from 86.4 (BLUEQ18) to 138.1 (BLUEC18) for GY, from 6.3 (BLUEAll) to 63.6 (BLUEQ17) for SPI, and from 4.8 (BLUEQ18) to 28.6 (BLUEQ17) for FE (Table 4, Fig. 3).

## Discussion

GS has been used to select superior genotypes in different plant breeding programs. It is being employed more now due to the availability and continuously reduced cost of advanced DNA sequencing techniques. In the past, ST-GS was a popular method to evaluate the performance of plant genotypes. However, plant breeders generally collect data for several traits for selection purposes, which provides an opportunity to use multiple traits in GS models. To determine whether incorporating physiological traits in the prediction model increases the predictive ability of traits of interest, we compared two MT-GS methods (MT-CV1 and MT-CV2) with ST-GS method (ST-CV1). In the ST-CV1, we evaluated the predictive ability of five primary traits (GY, HI, GN, SPI, FE) individually. In MT-CV1 and MT-CV2 models, we included CT and NDVI as secondary traits along with five primary traits.

ANOVA data showed significant genotypic and environmental effects. The Genotype-by-environment effect was not significant for NDVI and CT. The larger influence of G × E on other primary traits resulted in a lower heritability as they are complex polygenic [37, 49]. CT serves as a proxy for stomatal conductance. Lower CT indicates favorable water status and transpiration rate under stress [26, 50], and also suggests superior root system, chlorophyll content, and membrane stability [26]. In this study, CT had a negative association with all the tested traits in all environments. Negative associations between CT and other traits such as GY, HI, and NDVI have been previously reported in wheat [26, 27]. NDVI is a rapid measurement of leaf greenness and chlorophyll content, which has been associated with higher abiotic stress tolerance, grain yield, and its components [26, 28]. We also found positive correlations between NDVI and HI, GY, GN, SPI, FE, and HI in this study.

MT-CV1 and ST-CV1 models showed similar predictive ability in most cases, consistent with many other studies [47, 48, 51]. This illustrates that MT models are not always better than the ST model. Contrastingly, a few studies showed improvement in predictive ability when highly correlated and highly heritable secondary traits were incorporated in the MT-CV1 model [36, 51, 52]. This result is, however, not applicable for complex polygenic traits [36]. A similar heritability between primary and secondary traits and a relatively small population (*n* = 236) used in this study might have limited the efficacy of MT-CV1.

The MT-CV2 model improved predictive abilities for all five primary traits in this study although the extent of improvement fluctuated across traits and environments, which agrees with previous reports [45, 47, 48, 51, 53]. The improvement in predictive ability in MT-CV2 depends on the heritability of the primary traits. When a primary trait has low heritability, and a secondary trait has high heritability, MT-CV2 can improve predictive ability significantly. It also depended on the correlations between the primary and secondary traits [36, 45, 47, 53]. There was a lower improvement in predictive ability between ST-CV1 and MT-CV2 for traits like FE and SPI, which could be attributed to the combination of weak correlations between these primary and secondary traits and their heritabilities. Lacking genetic information on weakly correlated traits has shown to result in little improvement in predictive ability [36, 48, 51, 52]. Studies have shown that a model that includes two correlated traits is superior to the models with a single trait [48] or three correlated traits [54, 55]. It is pragmatic to use only few highly heritable, strongly correlated secondary traits to predict primary traits since incorporating many traits could add collinearity issues [48, 51, 54, 55]. Additionally, phenotyping too many traits costs breeding programs more money, time, and labor [48]. Furthermore, we also need to consider different factors such as marker density, QTL number, training population size (sample size), population structure, and relatedness among individuals in the training and testing population [1, 15].

Phenotyping some traits are more expensive, time-consuming, and labor-intensive than others, which makes implementing GS for these traits burdensome. The GS becomes cost-efficient when phenotyping of primary traits is more expensive and difficult than secondary traits. In this case, we only phenotype the training set for primary traits, but both training and testing sets for secondary traits. For instance, the MT-CV2 model resembles a scenario in a breeding program where physiological data are taken when plots are yet to be harvested in a later stage [55]. This could be particularly useful for traits like HI, GN, SPI and FE which are extremely labor and time intensive undertaking. Our study also found multi-trait model that used both CT and NDVI in general had better prediction accuracy for those traits compared to model that used a single trait, i.e. either CT or NDVI, with a few excptions (Supplementary file S1). NDVI and CT are easy to phenotype and their data are collected by different wheat breeding programs. Recently, plant breeders are utilizing high throughput phenotyping (HTP), including unmanned aerial vehicles (UAVs), to collect phenotypic data. With the increased use of UAVs, NDVI and CT can be measured simultaneously in a relatively short time in large number of genotypes. The constraint to use an MT model could be its complexity and need for high processing capability [36, 48, 51].

## Conclusions

To exploit genetic information from correlated traits using an MT-GS method, GS using two traits could be useful to improve the genomic prediction accuracy of a primary trait of interest. In a wheat breeding program, physiological traits such as CT and NDVI are measured routinely to evaluate stress tolerance along with other agronomic traits. We compared predictive ability among ST prediction model (ST-CV1) and two MT genomic prediction models (MT-CV1 and MT-CV2) and found that the phenotypically correlated secondary traits in both the training and testing sets (MT-CV2) improved predictive ability giving the high correlation between primary and secondary traits. Whereas improvement in predictive ability was not obvious when the secondary trait was incorporated only in the training set (MT-CV1). This result is highly useful in breeding programs where data for several traits are usually collected. Multi-trait genomic selection involves measuring laborious and expensive traits in a smaller training population, whereas phenotyping of inexpensive correlated traits in the testing population. With the increasing availability of the HTP platforms, the MT-GS methods can facilitate improvement in the genetic gain for many important traits in wheat.

## Methods

### Materials and experimental design

The genotypes used in this study consisted of 236 facultative soft wheat elite lines and varieties that were developed by different wheat breeding programs in the south and soueastern USA (Texas A&M, Virginia Tech, University of Georgia, University of Arkansas, North Carolina State University, Louisiana State University, University of Kentucky, and University of Maryland). The wheat lines used in the present study are mostly facultative in nature and vernalization requirements are generally low and are well adapted to the warm and humid southern and southeastern regions of the USA. The field experiments were carried out in two locations: Plant Science Research and Education Unit (PSREU) in Citra, Florida in 2017–18 growing season and North Florida Research and Education Center (NFREC), Quincy for two growing seasons (2016–17 and 2017–2018). An augmented design was used with three repeated check varieties (SS8641, PI 674197; AGS2000, PI 656845; Jamestown, PI 653731) that are widely grown wheat in the southern and southeastern US to control spatial variability. The size of six-row plot used for.

the study was 5.1 m^2^ (3.33 m long/1.52 m wide) with a seed rate of 100 kg ha^− 1^. Management and agronomic practices such as fertilizer and chemical application and irrigation were performed as recommended for optimum growth and yield potential. Fungicides were sprayed as needed at stem elongation, booting, and early grain filling to prevent different foliar and spike diseases. The weather data is listed in Table 5.

**Table 5:** Weather table showing T_ave_ (monthly average temperature) and Ppt (monthly precipitation in mm). The wheat panel was planted for two seasons in Citra (2017/ 2018) and Quincy (2016/2017, 2017/2018)

Month/ Year	Citra (2017–2018)		Quincy (2016–2017)		Quincy (2017–2018)	
	T _Ave_(°C)	Ppt (mm)	T _Ave_(°C)	Ppt (mm)	T _Ave_(°C)	Ppt (mm)
11/16	18.06	78.49	16.09	10.16	15.35	11.18
12/16	14.86	40.64	14.54	134.37	12.10	80.77
01/17	10.84	132.84	13.74	237.49	8.14	52.32
02/17	19.85	63.75	16.03	74.68	17.40	133.86
03/17	16.10	80.26	16.62	31.50	14.57	137.67
04/17	20.35	170.69	20.19	86.87	17.99	67.56
05/17	24.03	205.49	22.66	151.13	23.56	205.99

### Phenotyping

Five primary traits (HI, GY, GN, FE, and SPI) and two physiological traits CT and NDVI were measured in the present study. Days to anthesis was taken for each plot as the days from planting to the day when 50% of plants were flowered [56]. At 7 days after anthesis (Zadoks scale: GS70), the plant sample was cut at ground level from 0.25 m^2^ area of each plot. The sample was oven-dried at 60 °C temperature for 72 h. The weight of the total dried sample was collected, and the fertile spike number was counted. Spikes and stems were separated, and weights were collected. Spike partitioning index was calculated as a ratio of total spike dry weight to the above-ground dry matter at anthesis plus 7 days. Traits such as GN, GY, and HI were recorded at physiological maturity (Zadoks scale: GS90). Days to physiological maturity for each plot was taken when the flag leaves and spikes turn yellow. Grain number m^− 2^ was calculated by dividing total grain weight by individual grain weight. Harvest index was measured as the ratio of grain weight m^− 2^ to total dry biomass m^− 2^. Likewise, GY (kg ha^− 1^) was measured as a total seed weight from each plot after adjustment with 12% moisture. FE was calculated as a ratio of GN (m^− 2^) at maturity and spike dry matter (m^− 2^) at anthesis plus 7 days. CT was collected at three growth stages, heading (H), mid-grain filling (MGF), and late-grain filling (LGF), between 1300 and 1500 h on sunny days when the temperature reached the daily high by using Fluke 572–2 IR thermometer (Fluke Corporation, Everett WA). CT data were collected from both sides of each plot at a 50 cm distance from the edge and approximately 50 cm above the canopy at an angle of 30^o^ to the horizontal. The mean value of two readings was calculated for each growth stage and the average of three values from the three growth stages was used for further statistical analysis. NDVI was measured at four growth stages: H, early-grain filling (EGF), MGF, and LGF using the GreenSeeker handheld crop sensor (Trimble Navigation Limited) by holding it 50 cm above the canopy facing the center of the plot. The mean value of those readings was used for statistical analysis.

### Genotyping

The genotyping method has been explained in detail in a previous paper [49]. We obtained 27,466 SNPs as a result of SNP calling and filtering. Missing values were imputed with the LD-KNNi method [57] implemented in TASSEL v.5. For genomic prediction models, SNPs were converted to − 1, 0, and + 1, where − 1 indicated minor allele at a given locus, 0 indicated heterozygous loci, and + 1 indicated major allele at a given locus. The additive relationship matrix (K) was estimated using the ‘A.mat’ function in the ‘rrBLUP’ package in R [17].

### Phenotypic data analysis

Analysis of variance (ANOVA) was conducted using the “lme4” package [58] in R software (v3.5.1, R Development Core Team). The best linear unbiased estimates (BLUEs) were obtained for three individual environments, Quincy 2016–2017 (BLUEQ17), Quincy 2017–2018 (BLUEQ18), and Citra 2017–2018 (BLUEC18), and a combined across environments (BLUEAll). All traits were adjusted using days to anthesis as a covariate. Two statistical models were used to calculate adjusted values following Lozada and Carter [59]. The models used were for individual environment was as follows:

$${\mathrm{Y}}_{\mathrm{i}\mathrm{jkl}}=\upmu +{\mathrm{Block}}_{\mathrm{i}}+{\mathrm{IDCheck}}_{\mathrm{j}}+{\mathrm{Gen}}_{\mathrm{k}}+{\mathrm{Check}}_{\mathrm{l}}+{\upvarepsilon}_{\mathrm{i}\mathrm{jkl}}$$

For combined analysis across environments, the statistical model was as follows.

$${\mathrm{Y}}_{\mathrm{ijklm}}=\upmu +{\mathrm{IDCheck}}_{\mathrm{j}}+{\mathrm{Gen}}_{\mathrm{k}}+{\mathrm{Check}}_{\mathrm{l}}+{\mathrm{Env}}_{\mathrm{m}}+{\mathrm{IDCheck}}_{\mathrm{j}}\times {\mathrm{Env}}_{\mathrm{m}}+{\mathrm{Gen}}_{\mathrm{k}}\times {\mathrm{Env}}_{\mathrm{m}}+{\mathrm{Check}}_{\mathrm{l}}\times {\mathrm{Env}}_{\mathrm{m}}+{\mathrm{Block}}_i\Big({\mathrm{Env}}_{\mathrm{m}\Big)}+{\upvarepsilon}_{\mathrm{ijklm}}$$

where Y is the phenotype of a trait of interest; μ is the effect of the mean; Block_i_ is the effect of ith block; Gen_k_ is the effect of kth genotypes; Check_l_ is the effect of the lth checks on each block; Env_m_ is the effect of the mth environment. IDCheck_j_ is the effect of jth IDCheck. IDCheck was used to differentiate the effects of one check over the other checks, as well as the number of checks present on each block; IDCheck_j_ x Env_m_, Gen_k_ x Env_m_, and Check_l_ x Env_m_ are the effects of check identifier by environment, genotype by environment, and check by environment interactions, respectively. Block_i_(Env_m_) is the effect of ith block nested within mth environment and ε is the residual.

Broad-sense heritability was calculated assuming genotype and other effects as random [59] and was obtained by:

$${H}^2=\frac{\upsigma_{\mathrm{G}}^2}{\upsigma_{\mathrm{G}}^2+\frac{{\upsigma^2}_{\mathrm{G}\times \mathrm{E}}}{\mathrm{n}}+\frac{\upsigma_{\mathrm{e}}^2}{\mathrm{n}\mathrm{r}}}$$

where *H* ^2^ is a broad-sense heritability estimate, ${\upsigma}_{\mathrm{G}}^2$ is genetic variance, σ^2^_GXE_ is genotype-by-environmental variance, ${\upsigma}_{\mathrm{e}}^2$ is residual variance, n is the number of environments, and r is the number of replications per environment (i.e. equal to 1 for an augmented experimental design).

Pearson’s correlation among traits was calculated from BLUEs in R using the “corrplot” package in R [60]. PC biplot was generated in R by using the “factoextra” R package [61]. Single and MT-GS models were used to evaluate various traits.

### Single trait (ST) model

**Fig. 4:**
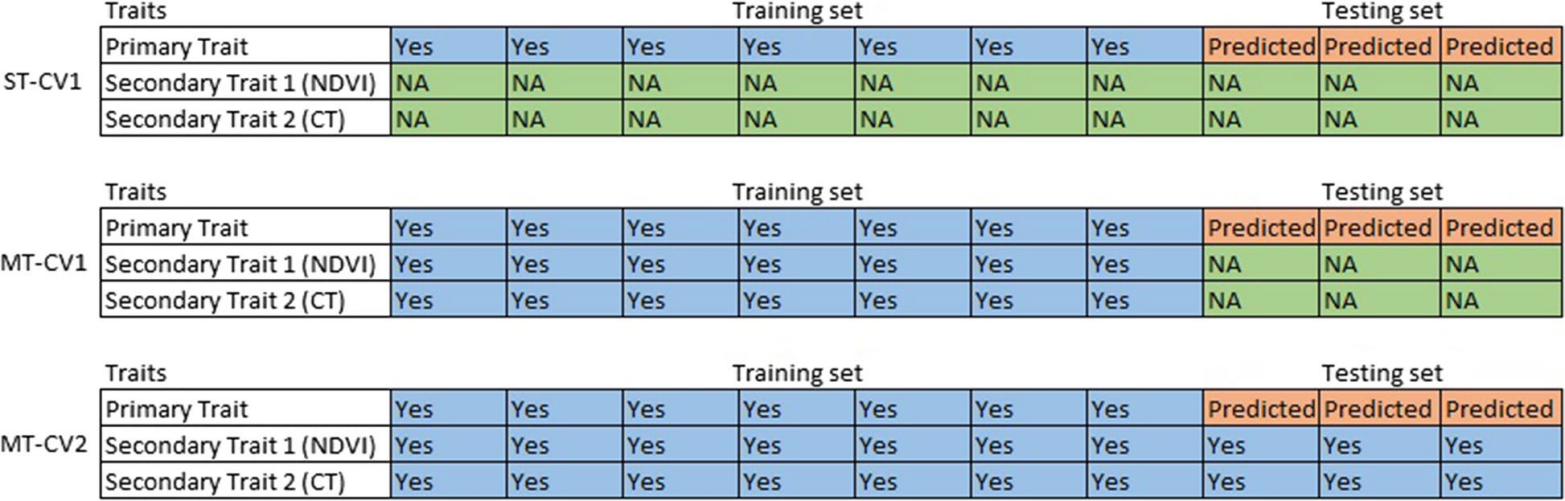
Cross-validation schemes employed. ST-CV1: single-trait cross-validation scheme where a training set of 70% of random genotypes are phenotyped and genotyped and a testing set of remaining 30% of genotypes are genotyped, not phenotyped; MT-CV1: multi-trait cross-validation scheme where a training set of 70% of random genotypes are phenotyped (primary + secondary traits) and genotyped and remaining 30% of genotypes are genotyped only, not phenotyped; MT-CV2: multi-trait cross-validation scheme where 100% information from secondary traits, a training set of 70% of random genotypes are phenotyped for primary traits and remaining 30% of genotypes as testing set (phenotyped for correlated traits but not primary traits and genotyped)

In the ST model, the prediction was obtained by using a Bayesian ridge regression (BRR) model with 2000 burn-ins and 12,000 iterations for the Gibbs sampler algorithm [48, 51] implemented in the ‘BGLR’ package [62] in R software. The following model was used.

$$\mathbf{y}=\boldsymbol{\upmu} +\mathbf{Z}\boldsymbol{\upalpha } +\boldsymbol{\upvarepsilon}$$

where **y** is the vector of BLUE values for a single trait; **μ** is the vector of the overall mean; **Z** is a design matrix with random marker effects, **α** is a genotypic predictor with **α** ~N(0, Kσ ^2^_g_) where **K** is the realized additive relationship matrix and σ^2^_g_ is additive genetic variance and **ε** is the residual errors vector with **ε** ~N(0, **I**σ ^2^_e_) where **I** is the identity matrix. Prediction accuracies were estimated using a cross-validation approach CV1 [63], explained in (Fig. 4).

### Multi trait (MT) model

The MT model was built using a Bayesian multivariate Gaussian model to estimate an unstructured variance-covariance matrix between traits (Σ) and residual matrix (R) with 2000 burn-ins and 12,000 iterations for the Gibbs sample algorithm [48, 51] implemented in the ‘MTM’ package [64] in R software using the model:

$$\begin{bmatrix}{\mathbf y}_{\mathbf1}\\\boldsymbol\vdots\\{\mathbf y}_{\mathbf t}\end{bmatrix}\boldsymbol=\begin{bmatrix}{\mathbf\mu}_{\mathbf1}\\\boldsymbol\vdots\\{\mathbf\mu}_{\mathbf t}\end{bmatrix}\boldsymbol+\begin{bmatrix}{\mathbf Z}_{\mathbf1}&\boldsymbol\cdots&\mathbf0\\\boldsymbol\vdots&\boldsymbol\ddots&\boldsymbol\vdots\\\mathbf0&\boldsymbol\cdots&{\mathbf Z}_{\mathbf t}\end{bmatrix}\begin{bmatrix}{\mathbf\alpha}_{\mathbf1}\\\boldsymbol\vdots\\{\mathbf\alpha}_{\mathbf t}\end{bmatrix}\begin{bmatrix}{\mathbf y}_{\mathbf1}\\\boldsymbol\vdots\\{\mathbf y}_{\mathbf t}\end{bmatrix}\boldsymbol{\mathit+}\begin{bmatrix}{\mathbf\varepsilon}_{\mathbf1}\\\boldsymbol\vdots\\{\mathbf\varepsilon}_{\mathbf t}\end{bmatrix}$$

where **y** is a vector of BLUE values for t traits; **μ** is the overall mean; **Z** is the incidence matrix; **α** is a genotypic predictor with **α** ~MVN (0, **Σ** ⊗ **K**) and **ε** is the residual errors vector with **ε** ~MVN (0, **R** ⊗ **I**), where **Σ** is the variance-covariance matrix across traits, **K** is the realized additive relationship matrix among individuals estimated from the markers, **R** is the variance-covariance matrix for the residual effects for each individual among traits, **I** is the identity matrix, and ⊗ is the Kronecker product of two matrices. **Σ** was estimated as an unstructured matrix and **R** as a diagonal matrix [48].

### Cross-validation (CV)

The Monte-Carlo cross-validation scheme was used to estimate prediction accuracy [48, 51] (Fig. 4). The CV1 scheme was applied to both ST and MT models (ST-CV1 and ST-CV1), respectively. The CV2 scheme was applied only in the MT model (MT-CV2).

### Cross-validation Scheme 1

The first cross-validation scheme (CV1) used a training set (TP) of 70% of random genotypes (*n* = 165) which have phenotypic (primary+secondary traits for MT-CV1) and genotypic data. The testing set (VP) consisted of the remaining 30% of genotypes (*n* = 71) that have genotypic data only. This process was repeated for 100 times, where each iteration included a different combination of genotypes in training and testing sets. Predictive ability was calculated as a mean of Pearson’s correlations between observed phenotypic values and predicted values.

### Cross-validation Scheme 2

The same as in CV1, the second cross-validation scheme (CV2) used the phenotypic and genotypic data from the training set of 165 lines. However, the genotypic data and phenotypic data of physiological traits from the testing set of 71 lines were used. In other words, the CV2 scheme not only used genotypic information from both TP and VP and phenotypic data of the primary traits (HI or GY or GN or SPI or FE) from the TP but also used phenotypic data of secondary correlated traits (NDVI and CT) from both TP and VP. This process was repeated 100 times, where each iteration included a different combination of genotypes in the TP and VP. Predictive ability was calculated as a mean of Pearson’s correlations between observed phenotypic values and predicted values.

## Supplementary Information

**Additional file 1.**

## Data Availability

The phenotypic datasets used and/or analyzed during the current study are available from the corresponding author on reasonable request. The genotypic datasets generated and/or analyzed during the current study are available in the NCBI using accession number PRJNA578088 (https://www.ncbi.nlm.nih.gov//bioproject/PRJNA578088).

## Abbreviations

GS: Genomic selection
MT: Multi-trait
CT: Canopy temperature
NDVI: Normalized difference vegetation index
HI: Harvest index
GY: Grain yield
GN: Grain number
SPI: Spike partitioning index
FE: Fruiting efficiency
CV1: Cross-validation Scheme 1
CV2: Cross-validation Scheme 2
MT-CV2: Multi-trait cross validation 2
ST-CV1: Single-trait cross validation 1
MT-CV1: Multi-trait cross validation 1
TP: Training population
VP: Validation population
GEBV: Genomic estimated breeding value
MAS: Marker assisted selection
GBS: Genotype-by-sequencing
rrBLUP: Ridge regression best linear unbiased prediction
GBLUP: Genomic best linear prediction
LASSO: Least absolute shrinkage and selection operator
BRR: Bayesian-based methods: Bayesian ridge regression
RKHS: Reproducing kernel Hilbert spaces regression
PT: Physiological traits
HTP: High throughput phenotyping
H: Heading
EGF: Early-grain filling
MGF: Mid-grain filling
LGF: Late-grain filling
BLUEs: Best linear unbiased estimates
BLUEC: BLUE values estimated from Citra
BLUEQ: BLUE values estimated from Quincy
BLUEAll: BLUE values estimated from all environments
